# Alternative views of biological species: reproductively isolated units or genotypic clusters?

**DOI:** 10.1093/nsr/nwaa116

**Published:** 2020-05-30

**Authors:** James Mallet

**Affiliations:** Department of Organismic and Evolutionary Biology, Harvard University, USA

Wang *et al*. in this journal argue that it may be time to abandon a classic idea about species, the biological species concept (BSC), given recent findings with genomic data on closely related taxa [1]. Furthermore, they propose a set of tests on genetic or genomic data that might lead to acceptance or rejection of the biological species concept.

Wang *et al*. are not the first to critique the biological species concept (BSC), which has survived an onslaught of attacks from many fronts. What is the biological species concept? And what is the new critique about? In this commentary, I briefly cover the history of ideas about biological species, then discuss current work that depends on rich, genome-scale sequence data, before attempting to resolve issues.

## THE BIOLOGICAL SPECIES CONCEPT

The term ‘biological species concept’ (BSC) was coined originally by Ernst Mayr, building on Dobzhansky's ideas of reproductive isolation [2–4] during the later phases of the ‘Modern Synthesis’ between Darwinian evolution and Mendelian genetics. Dobzhansky and Mayr argued that species are best viewed as reproductively isolated units: ‘Species are groups of actually or potentially interbreeding natural populations, which are reproductively isolated from other such groups’ [3,4]. Reproductive isolation was viewed as a set of ‘mechanisms’ that prevent species from merging: ‘each species is a delicately integrated genetic system … Hybridization would lead to a breakdown of this system and would result in the production of disharmonious types. It is the function of the isolating mechanisms to prevent such a breakdown, and to protect the integrity of the genetic system of species.’ (ref. [4], p. 109). ‘There is a tendency in the integrated gene complex to establish an ever greater cohesion, to achieve a steady improvement of developmental and of genetic homeostasis…,’ and ‘Speciation is potentially a process of evolutionary rejuvenation, an escape from too rigid a system of genetic homeostasis’ (ref. [4], p. 555).

Associated with these views there exists a related set of ideas that species are more ‘real’ in some way than lower taxa such as geographic races, or higher taxa such as genera: ‘The evolutionary significance of species is now quite clear. … The species are the real units of evolution, as the temporary incarnation of harmonious, well-integrated gene complexes’ (ref. [4], p. 621). Under this new, mid-20th Century view of species, speciation was a special kind of evolution distinct from other modes of evolution at the population level, and Darwin was criticized as having not really dealt with speciation at all in his book ‘On the Origin of Species…’ [5]. According to Ernst Mayr, ‘Darwin's failure… resulted to a large extent from a misunderstanding of the true nature of species.’ (ref. [4], p. 14). A few scientists today still agree with these criticisms of Darwin's ideas about speciation [6,7].

Although the resultant BSC is a very successful and useful-seeming meme, the idea that species are best defined as reproductively isolated units was not without critics. An underlying theme was that the sharp discontinuities built into the BSC conflicted with the idea of speciation as a gradual, emergent Darwinian process. Mayr clearly recognized this: in fact, as we have seen, he believed that gene flow and the lack of reproductive isolation among populations hindered evolutionary progress and prevented speciation. In his view, speciation was ‘complete’ when there was a complete absence of successful gene flow (see also ref. [8]). In a sense, by requiring almost complete reproductive isolation between species, and a complete lack of it within species, speciation became an almost insurmountable barrier for gradual Darwinian evolution to bridge.

Mayr's solution to this difficulty was the proposition that populations would normally diverge into separate species while geographically isolated, in ‘allopatry’ [3]. However, gradual vicariant divergence was acknowledged to be rather slow, maybe too slow to generate the diversity of species we observe on our planet today. To solve the issue of the slow rate of divergence demanded by his view of species as optimized ‘delicately integrated genetic systems,’ Mayr popularized another idea, that small populations dispersing to geographically separated locations could speciate more rapidly by means of the ‘founder effect’, leading to a ‘genetic revolution’ via rapid reorganization of coadapted gene complexes [4,9]. Today the founder effect is deemed poorly supported by theory or data, while the more general idea that allopatric speciation is the norm still holds some sway [6,10–12].

## PROBLEMS WITH THE BIOLOGICAL SPECIES CONCEPT

A major issue with the BSC concerns its supposed role in evolution. Ehrlich and Raven [13] pointed out that local populations, rather than species, were the actual foci of evolution, and that whether or not such populations belonged to a widely-distributed biological species had little to do with their local evolution, since gene flow was evident mostly on a local, rather than a global scale within widespread biological species. Instead, ‘selection itself is both the primary cohesive and disruptive force in evolution; the selective regime determines what influence gene flow has on observed patterns of differentiation. Populations will differentiate if they are subjected to different selective forces and will tend to remain similar if they are not’ [13]. Gene flow undoubtedly constrains divergent natural selection, but only on a very local scale. Populations will diverge over spatial scales >*σ*/√*s*, where *σ* represents gene flow distance, and *s* represents the strength of divergent selection on a gene. Therefore, populations will diverge, even under relatively weak selection (say *s* ∼ 0.01), on a local spatial scale of a only handful of gene flow distances [14,15].

The BSC appears to depend on a rather binary view of reproductive isolation (either on: yes – a species; or off: no – not a species), it is not operational in taxonomy, especially for far-flung local populations not in contact, where under Mayr's rubric, one would need to assess whether the populations were ‘potentially interbreeding.’ Sokal and Crovello [16] ‘having decided that the BSC is neither operational nor heuristic nor of any practical value,’ concluded that a more operational ‘phenetic species as normally described is the desirable species concept to be associated with the taxonomic category ‘species,’ and that the localized biological population may be the most useful unit for evolutionary study.’ Their argument for ‘phenetic’ species was a statisticians' version of Darwin's own argument that species were best defined as clusters of individuals separated from other such groups by gaps in the distribution of morphologies (e.g. ref. [5]: pp. 58–9). Like Ehrlich and Raven, Sokal & Crovello [16] recognized instead local populations, rather than species, as the main focus for evolution.

Others found different problems. Oak species had been long recognized, but many hybrids were also known to exist naturally. In spite of gene flow, distinct ecologically associated lineages, or species, of oak trees remain identifiable, presumably due to divergent ecological niches. Ecological divergence, rather than reproductive isolation, defined such species [17].

Hugh Paterson pointed out that protection of species from introgression as a function of isolating mechanisms under the BSC was an unlikely and anti-Darwinian idea: natural selection tends to safeguard the individual's interests, not those of its population or species. Species, Paterson suggested, should be defined by mate recognition within species, and not via protection from invasion by other species [18,19]. We may agree that the idea that reproductive isolation functions to protect species integrity under Mayr's BSC was group selectionist, but it is arguable that ‘mate recognition’ is not a function of sexual behavior at the level of species as Paterson supposed. Instead, individuals mate to maximize their own individual fitness, and so the idea of mate recognition at the species level is almost as prone to a group selection fallacy as reproductive isolation.

If species evolve from geographic populations, then they should often pass through earlier phases deemed by Ernst Mayr and others under the BSC to be geographical subspecies. However, many named subspecies have unique trait combinations. Cracraft suggested that these ‘tips’ of the phylogenetic tree should be classified as separate species [20]. In his view, species are populations with consistently different character combinations. Any diagnosable population could be considered as a separate species under Cracraft's phylogenetic species concept (PSC), regardless of reproductive compatibility, provided that the members of such populations showed ‘a parental pattern of ancestry and descent.’

The fact that many nominal species, especially in plants such as oak trees, engaged in frequent hybridization (‘too much sex’) and that other species were asexual (‘too little sex’), and yet were still classified as species led to the idea that factors other than reproductive isolation combined to cause the cohesion of species, in Templeton's ‘cohesion species concept.’ Ecological factors and even genealogical relationships that help maintain separate species status were included as mechanisms of cohesion in addition to classical reproductive isolation [21,22]. And there were many other critiques, especially a variety of other conflicting species concepts based on phylogenetic principles, as well as a species concept based on monophyly of gene genealogies [23].

In spite of all these criticisms, many of today's evolutionary biologists still maintain that the BSC is a reasonable definition of species. Of course, ‘reproductive isolation,’ today often called ‘reproductive barriers,’ does in a sense play a role in keeping taxa that we call species apart. Therefore, to understand speciation in sexual species seems to require investigating something like reproductive isolation so that a pair of taxa can diverge into separate species that can coexist spatially. Thus the BSC has survived as one of the more enduring ideas about species, with many modern texts finding it more useful for discussions of evolution and speciation than alternatives [6,12,24]. The study of speciation therefore appears to be equivalent to a goal of understanding the evolution of reproductive isolation [6]. But the goal all along was to understand how populations could diverge sufficiently to allow coexistence in sympatry [4,25].

## SPECIES AS GENOTYPIC CLUSTERS, 25 YEARS ON

My own paper arguing for a multi-locus ‘genotypic cluster’ definition of species, updating Darwinian ideas on species with genetics, has its 25^th^ anniversary this year [26]. Our studies on ‘host races’ in the larch budmoth and hybridization among species and subspecies of *Heliconius* butterflies [27–31] led me, as with Van Valen and Templeton earlier [17,22], to a view that species were often distinguishable in spite of continued gene flow in sympatry. Factors generally termed reproductive isolation were indeed among the *causes* of speciation, but they were not very helpful in the *definition* of species. Attempts have been made to justify a weaker version of the BSC than Mayr's to allow for gene flow after speciation (e.g. ref. [6]), but it is unclear how much gene flow would be allowed under this idea. Instead of Templeton's tactic to include within ‘cohesion mechanisms’ all possible diverse processes that prevent species from fusing in the definition of species, I argued that we could more simply define what we were talking about operationally, using the pattern of observable traits and genetics of those taxa that did not fuse in sympatry [26].

Having realized that species evolve rather than were created, it was obvious to Darwin that species were going to be less ‘real’ than hitherto assumed. What Darwin meant by species in ‘The Origin of Species’ were populations separable because of morphological gaps between them [5]. In population genetic terms, the equivalent is that we recognize species when there is a deficit of heterozygotes at loci that differ, or more powerfully, when there is a deficit of recombinants among such loci. In modern terms, species are recognized operationally by the gaps between their genetic traits (deficits of intermediates and recombinants) when they overlap in space and time. Strong correlations among allelic differences between populations (linkage disequilibrium), is the key marker of species [26]. This Darwinian definition of species is agnostic to the precise processes, such as the reproductive isolation or ecology that led to or maintain separateness, while not denying that reduced gene flow is important.

In genetic terms, Darwin's idea of species can be associated with the idea of species as genotypic clusters [26]. Others working on the tractable host-plant races of the apple maggot *Rhagoletis pomonella* had come to similar conclusions at the same time [32]. The idea of species as genotypic clusters derived from a viewpoint similar to that of Sokal & Crovello [16], but with a more explicit population genetics focus, for example on linkage disequilibrium (a deficit of recombinants among differentiated alleles within individual genomes). I called this ‘A Species Definition for the Modern Synthesis,’ because I was concerned to justify existing taxonomic use of the BSC, which I knew well from the Lepidoptera I studied, and which seemed usefully to employ a sympatry-based species delimitation: parapatric subspecies that blended freely at their contact zones would be included within the same species.

In contrast, many of the geographic subspecies that we studied could have been divided into multiple separate species under Cracraft's [20] ‘diagnostic’ version of the phylogenetic species concept (PSC): this seemed to me to lead to unnecessary levels of taxonomic splitting. My minimalist goal was to ensure that divergent clusters of genotypes would be classified as separate species in sympatry, and to suggest operational methods (e.g. studying the genetics of clusters of genotypes and phenotypes in sympatry), rather than to employ the inferential lip service to reproductive isolation as demanded by the BSC [26].

## THE GENIC VIEW OF SPECIATION

Meanwhile, Chung-I Wu and his group had been investigating ‘speciation genes,’ the loci that contribute to hybrid sterility and inviability, in *Drosophila*. As short-fragment DNA sequencing became accessible due to advances in molecular genetics, Wu's group found that a ‘speciation gene,’ *Odysseus* (*OdsH*), had a genealogy among *D. mauritiana*, *D. sechellia*, and *D. simulans*, that matched a likely speciation tree. However, loci as little as 1.8 kb distant from this gene yielded a different genealogy (i.e. gene tree), suggesting that gene flow beyond species boundaries could have occurred after acquisition of *Odysseus*-related reproductive isolation [33]. These findings led Wu [34] to the idea that ‘speciation is the stage where the gene pools at loci of differential adaptation (i.e. genes that cause speciation) would not mix even when the extrinsic barriers to gene exchange are removed and, furthermore, will be able to continue to diverge. Thus, the very essence does not have to include reproductive isolation.’

Wu saw reproductive isolation and speciation as processes that happened to genes, rather than to the whole genome as in Mayr's view. His ‘genic view of speciation’ therefore allowed parts of the genome not involved in divergent selection or hybrid incompatibility to flow between species, even after an irreversible stage of speciation had been reached [34,35]. In that this genic view allowed gene flow among species, the concept of species was similar to that of Mallet [26], except that the ‘genic view of speciation’ stressed that the means of divergence was reproductive isolation due to ‘speciation genes.’ In contrast, Mallet's genotypic cluster criterion was agnostic as to means of divergence [26].

## THE VIEWS OF WANG *ET AL*. IN 2020

In their new article, Wang *et al*. [1] regard the BSC as ‘demanding a prolonged period of divergence during which there is no gene flow.’ The ‘salient feature’ of the BSC, according to Wang *et al*., is that ‘a period of strict geographical isolation (or allopatry) … is needed to complete the process of speciation’ [1]. Although Mayr and others have regarded speciation as largely occurring via allopatry, and although, as I have pointed out above, allopatry is perhaps more useful for speciation under the BSC than for many alternative species concepts (because the BSC was perceived as a stable system incapable of diverging in the face of gene flow), supporters of the BSC today do not preclude the possibility of sympatric or parapatric speciation via their species concept.

Wang *et al*. then argue that early in the process of speciation, during the initial evolution of reproductive isolation, there is likely to be some gene flow, even under the BSC model [1]. But subsequently, the BSC allows for no introgression whatsoever (Part A of their Fig. 1), according to Wang *et al*. In contrast, under an alternative model, a concept closer to Wu's genic view of speciation (part B of their Fig. 1), ‘gene flow continues, possibly diminished, all the way to the completion of speciation when traits of reproductive isolation have evolved.’

To test their genic view, Wang *et al*. carry out simulations of genomes affected by gene flow, with a few ‘speciation genes’ under divergent selection, but separated by many neutral loci that can be recombined away from these. They show that under some combinations of parameters, it is possible to generate genomes in which genomic regions near the speciation genes remain diverged, while other intervening loci recombine relatively freely among the species, and get swamped by introgression. This is strongly reminiscent of the ‘islands of speciation’ idea, as found between *Anopheles gambiae* and *A. coluzzii* (formerly called the M and S strains of *A. gambiae*) [36].

## GENE FLOW: EARLY OR LATE IN SPECIATION?

There are now abundant genomic data on the question of continuing gene flow for many of the taxa that we call species. Wang *et al*. [1] argue that the existing data may allow us to test ‘between the genomic [i.e. BSC] and genic view of speciation.’ In the canonical BSC view, the authors argue, gene flow is allowed in the very early stages of speciation, when geographic isolation is incomplete, but thereafter a prolonged period of geographic isolation is needed to ‘complete’ speciation. In contrast, under the alternative genic view of speciation, according to Wang *et al*., allopatry is not required, and gene flow can continue right up to ‘completion’ of speciation, and indeed maybe long afterwards, until finally, reproductive isolation is complete (i.e. gene flow is negligible).

But what is early gene flow, and what is late gene flow? Are divergent populations species before ‘reproductive isolation is complete?’ Recent genomic data show that gene flow occurs between species that are millions of years apart, and between non-sister species. The authors review existing literature for gene flow among named species, and while acknowledging its existence, they say ‘we find no case of large scale introgression in late stages of speciation, when postmating reproductive isolation is evident.’ Instead, all or at least most existing examples of introgression are listed by Wang *et al*. [1] as ‘early stage events.’ To take an example, they review the data on the *Anopheles gambiae* group. As well as abundant evidence for genomic islands of speciation and gene flow between *A. gambiae* and *A. coluzzii* [36,37], more recent genomic data have shown that gene flow between non-sister *A. arabiensis* and *A. gambiae* lineages has affected ∼98% of the genome, causing the average gene tree today to differ from the inferred original branching order of species [38–40]. The view that the average gene tree in the *Anopheles gambiae* group of mosquitos is distorted away from the species tree is agreed on by all three sets of authors, in spite of the fact that the precise inferred species tree differs among all of these three papers based on the same data! Nonetheless, Wang *et al*. argue that ‘it is evident that the introgressions in these species are early-stage events’ [1], and therefore that these observations do not constitute proof against the BSC. However, if multiple species, especially non-sister species, are exchanging large fractions of their genomes, as in the *Anopheles gambiae* group, surely some of this is ‘late’ in the speciation process!

## AN ATTEMPT TO RESOLVE THE ISSUES

As we have seen, there is a lot to agree with in the ‘genic view of speciation’ and its critique of the BSC, as shown by the obvious overlap with my own view of species as ‘genotypic clusters’ between which gene flow is possible. However, the ‘genic view’ as propounded here suffers, in my view, from a reiteration of some major difficulties associated with the BSC. For example, the current article repeatedly mentions the ‘completion of speciation.’ While, in one of the authors' stated views, speciation need not necessarily preclude a trickle of continued gene flow [34], ‘complete speciation’ in the new paper seems to mean total cessation of gene flow that would affect the whole genome [1]. Arguably, in nature, speciation is not like that. There is in fact a continuum of gene flow that declines but still occurs, at least in some parts of the genome, long after taxonomists and evolutionary biologists generally recognize species. In the simplest ‘light-bulb failure’ model, there is a linear, albeit stochastic accumulation of incompatible substitutions with time, and species compatibility declines exponentially [41]. If epistasis is involved, then compatibility loss may speed up over time, compared to exponential decline, as in the ‘snowball’ model [8,42]. However, the compatibility predicted under all such idealized probabilistic models never actually reaches zero [41].

Empirical results bear this out. The hybrid between horse and donkey, the mule, has long held an almost iconic status as a completely sterile hybrid between species, and at least since Buffon [43] has supported the view that horses and donkeys are biological species that lack any capability for mixing. However, mules do occasionally have offspring, and this has been known at least since the 18^th^ century [44,45]. This potentially leads to occasional gene flow between horses and donkeys and vice-versa. Today, we can examine their genomes, which show a strong signal of introgression between the horse lineage and the donkey + zebra lineages of *Equus* [46].

*Heliconius* butterflies provide further examples: occasional gene flow continues at every step of the way between *H. cydno/timareta* and *H. melpomene*, in spite of a lack of ‘islands of divergence’ in the genome between these two lineages. While male hybrids are fertile, female hybrids are usually sterile, an example of Haldane's Rule, so that there is strong evidence of ‘postmating isolation’ between these two lineages [47]. However, we have now found a pair of species in widespread sympatry, *H. pardalinus* and *H. elevatus*, that show evidence for substantial introgression, leading to large patches of near panmixia across the genome, with occasional genomic islands of divergence presumably containing divergently selected speciation genes [48], very similar to the pattern shown by Turner *et al*. for *Anopheles* ‘islands of speciation’ [36].

As with *Anopheles*, we also find evidence for massive gene flow among non-sister species deep in the estimated species tree of the *Heliconius erato* group of species, and this again causes distortion of the average gene tree away from the presumed species tree [49]. Here, as well as in *Anopheles*, and in the *Heliconius cydno/timareta* and *H. melpomene* hybridization, gene flow results even though ‘postmating reproductive isolation is evident’ (a requirement of ref. [1] to reject the BSC). Wang *et al*. [1], in contrast, suggest that ‘speciation genes’ that resist introgression may not yet have developed in the cases of gene flow we have discovered between *Heliconius* species, which they argue means that this gene flow is taking place early in speciation (their Table 1), and that therefore does not refute the BSC.

Provided Wang *et al*. did not intend their BSC rejection criterion to be utterly tautological (because requiring that gene flow occurs, when it is completely prevented by ‘speciation genes,’ seems impossible), then *Heliconius* provides a counter-example. Contrary to Wang *et al*., the existence of frequent Haldane's Rule postzygotic hybrid sterility in *Heliconius*, as well as strong assortative mating [47,48,50] together demonstrate evolution of considerable prezygotic and postzygotic isolation. Furthermore, positive correlation of introgression probability with recombination rate along the *Heliconius* genome in a recent study suggests that there are actually a very large number of ‘postmating reproductive isolation’ loci that select against introgression, and that these loci are widely scattered across all 21 chromosomes [49].

The BSC versus the ‘genic view’ therefore seems hard to test, at least using the reasoning of Wang *et al*. [1]. In contrast, under a more operational genotypic cluster approach, species are usefully defined as able to maintain their distinctness at multiple unlinked loci (not, of course, at all loci, so allowing for introgression of some parts of the genome) while in sympatry with sister species. Today, a test of sympatric species status could be carried out using multiple genetic markers and a Bayesian assignment test such as STRUCTURE [51,52]. It is even arguable that Dobzhansky and Mayr meant something like this sympatry coexistence criterion when they proposed the BSC and coined ‘reproductive isolating mechanisms’ as the key. In the 1930s–1950s the unavailability of genetic markers would have led them to propose a heuristic, ‘reproductive isolation’ instead of, say, a lack of recombinants (in modern terms, strong linkage disequilibria) among unlinked marker loci in sympatry, when defining species.

If we instead adopt a sympatric genotypic cluster criterion, speciation can occur much earlier than in the ‘completeness’ view of speciation espoused by Wang *et al*., and we no longer need to deal with the problem of whether the gene flow is early or late during the ‘process of speciation.’ It may occur at any time after speciation, albeit normally with diminishing probability over time since the split between a pair of species.

## CONCLUSION

Wu's ‘genic view of speciation’ [34] originated in order to accommodate the possibility of gene flow after speciation, while also recognizing that reproductive isolation was involved in speciation. However, Wang *et al*. [1] show that it is difficult to test genomic data under this view against the alternative of the biological species concept. The reason for the difficulty of their test is that it is hard to know when speciation is ‘complete’ under either set of ideas. With species as genotypic clusters, the completeness of speciation is dictated by overlap in sympatry. What we call species in sexual taxa are merely populations that can overlap spatially without fusing (and this was certainly one the original intentions of the biological species concept).

Under all three views outlined above, reproductive isolation and divergent selection are probable causes of speciation in sexual species worth investigating. However, it would in my view be helpful to avoid including these processes in the definition of the taxa we call species. It is becoming especially difficult to understand what supposedly ‘reproductively isolated species’ are when we know, as in both *Anopheles* and *Heliconius*, that gene flow is so pervasive as to re-write the average history of their genes towards an entirely different set of topologies from the original species tree. It is helpful to separate cause and effect, and therefore, to focus on maintenance of divergence of at least some unlinked loci in sympatry as the most useful criterion of species.
